# Subcarrier Frequency-Modulated Continuous-Wave Radar in the Terahertz Range Based on a Resonant-Tunneling-Diode Oscillator

**DOI:** 10.3390/s20236848

**Published:** 2020-11-30

**Authors:** Adrian Dobroiu, Yusuke Shirakawa, Safumi Suzuki, Masahiro Asada, Hiroshi Ito

**Affiliations:** 1 Tokyo Institute of Technology, Meguro, Tokyo 152-8552, Japan; shirakawa.y.aa@m.titech.ac.jp (Y.S.); suzuki.s.av@m.titech.ac.jp (S.S.); asada@pe.titech.ac.jp (M.A.); 2 Center for Natural Sciences, Kitasato University, Sagamihara, Kanagawa 252-0373, Japan; h.ito@kitasato-u.ac.jp

**Keywords:** terahertz-wave radar, resonant-tunneling diode, frequency-modulated continuous-wave radar

## Abstract

We introduce a new principle for distance measurement in the terahertz-wave range using a resonant-tunneling-diode (RTD) oscillator as a source at 511 GHz and relying on the frequency-modulated continuous-wave (FMCW) radar technique. Unlike the usual FMCW radar, where the sawtooth frequency modulation is applied to the carrier, we propose applying it to a subcarrier obtained by amplitude modulation; this is advantageous when the source cannot be controlled precisely in oscillation frequency, but can easily be modulated in amplitude, as is the case of the RTD oscillator. The detailed principle and a series of proof-of-concept experimental results are presented.

## 1. Introduction

The interest in the terahertz radiation has seen an explosive growth in the past few decades, as proved by a range of metrics: the number of publications and patents on the subject; the number of researchers involved in the field; the number of companies offering terahertz-wave-related products and services; as well as the variety of applications that have already been found in industry, homeland security, safety, agriculture, medicine, communications, and so on [1].

Among the needs the terahertz radiation can address is that of measuring distances along its direction of propagation, also called *ranging*. The advantages of using terahertz waves for such a measurement are multiple: compared to microwave and millimeter wave ranging, the size of the smallest detectable objects is smaller, as it is proportional to the wavelength; additionally, for a similar reason, the size of the optics used in the terahertz range is relatively small; on the other hand, compared to infrared and visible light systems, terahertz waves have a greater ability of passing through degraded visual environments—smoke, fog, dust, etc.—as well as through a wide range of packaging materials: paper and cardboard, most plastics, fabrics, wood, etc. A number of ranging techniques exist that use the terahertz waves, such as measuring the flight time of pulses using time-domain spectroscopy [2], applying the frequency-modulated continuous-wave (FMCW) radar technique [3,4,5], or using interferometry [6].

To make terahertz-wave systems more easily usable in real-world applications, the components used in such systems need to have several practical properties, such as small size and weight, low power consumption, room-temperature operation, affordable cost, sufficient reliability, long lifetime, reduced complexity, and ease of integrating in a system. This has been particularly a great challenge for the source of the terahertz-wave radar, as many of the sources available until now have failed in meeting several of these demands. We believe a promising candidate for possibly becoming the most advantageous terahertz-wave source could be the resonant-tunneling diode (RTD) [7]. An RTD oscillator is smaller than 1 mm in all three dimensions, operates at room temperature, requires a DC bias voltage in the order of 1 V of which it draws a current below 100 mA, and produces a continuous terahertz wave with a frequency that can be selected at the fabrication time in a range up to the current record of 1.98 THz [8], although the emission frequency can also be adjusted to some extent by tuning the bias voltage. Its output power is currently in the 10 µW order for single elements and just below 1 mW for large arrays of RTD’s [9], but ongoing research is expected to improve that significantly, potentially by two or three orders of magnitude [10,11].

In recent reports [12,13], we described a new terahertz-wave radar technique based on the principle of the amplitude-modulated continuous-wave (AMCW) radar, which we verified experimentally using an RTD oscillator as the terahertz-wave source. While that radar system is able to measure absolute distances with a precision below 100 µm by determining the phase of the modulation signal at two modulation frequencies, it has one particularly limiting disadvantage: it can only measure one distance at a time. This can be sufficient in certain applications, such as the 3D imaging of metallic (or otherwise opaque and reflective) surfaces, but there are circumstances where multiple overlapping or adjoining surfaces in a scene need to be detected and ranged simultaneously. For example, in a security gate body scanner scenario, the individual layers of clothing on a person may need to be detected separately. Similarly, in various levels of vehicle-driving automation, from the warning and assistance of a human driver to the fully automated self-driving, the distances to a multitude of reflecting surfaces in the surrounding space need to be measured. In situations like these, the AMCW radar is no longer an option, which motivated us to look for other radar principles.

The obvious choice is the FMCW radar. In this type of radar, the oscillation frequency is swept linearly; the wave returning from the target is delayed relative to the local oscillator, and mixing the two generates a beat signal whose frequency carries information on the propagation time to the target and back. When the target has multiple parts located at different distances from the radar, each of them produces a different delay, and hence, a different beat frequency; by Fourier analysis of the beat signal, those distances are determined simultaneously.

The RTD oscillator does allow the modulation of its oscillation frequency to some extent, by adjusting the bias voltage. Moreover, by using variable capacitance diodes in the RTD oscillation circuit, the range of oscillation frequencies can be extended significantly by applying a separate voltage on the diodes to adjust their capacity and alter the resonance frequency of the circuit; an RTD source with an array of varactor diodes was reported to have a tuning range as wide as 320 GHz, from 580 to 900 GHz [14]. Unfortunately, we still do not have the technology to control the oscillation frequency of the RTD oscillator as precisely as required by the FMCW radar principle. The main obstacle appears to be the fact that even a small amount of terahertz radiation returning into the RTD resonance circuit strongly disturbs the oscillation and changes the generated frequency. A good optical isolator might solve the problem, but even then, the nonlinear relationship between the RTD bias voltage (and the varactor diode voltage, if used) and the oscillation frequency is complex, and precisely generating the desired frequency will still likely be challenging.

In this paper, however, we propose a totally different approach for applying the FMCW radar principle to a terahertz-wave radar based on an RTD source. Instead of modulating the RTD oscillation frequency, we modulate the amplitude, thus producing a subcarrier with a frequency in the gigahertz order; we then sweep the frequency of that subcarrier linearly, as needed for applying the FMCW principle. We call this the *subcarrier FMCW radar*. We are not aware whether a subcarrier FMCW radar technique has ever been attempted before, including outside the terahertz range.

The subcarrier can be produced easily by using commercially available signal generators able to produce frequency sweeps; the modulation signal is simply added to the RTD bias voltage through a bias tee. The fact that the RTD carrier frequency itself changes to some degree because of the bias voltage variations is not relevant, since the carrier is removed at detection. It is, however, important that the carrier is in the terahertz-wave range: the diffraction, reflection, scattering, and any other optical processes that the radar beam undergoes are determined by the carrier frequency and wavelength, and as such the optical qualities of the radar, including the beam diameter required to achieve a specific lateral resolution (when using focused beams), are given by the terahertz-frequency carrier, not by the gigahertz-range subcarrier.

This paper includes results previously reported at two conferences [15,16] as well as a large amount of information never published, such as the theoretical background, a series of improvements of the method, new measurements, and a thorough discussion of the results.

## 2. Principle

**Figure 1:**
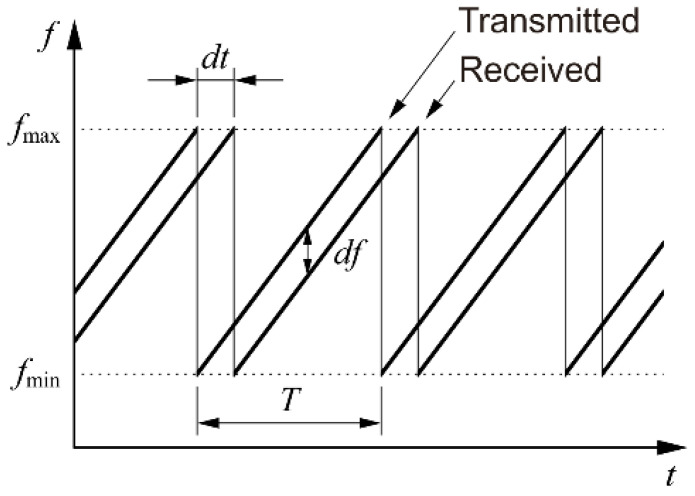
Principle of the FMCW radar. In this plot, the delay dt is exaggerated for the sake of clarity; in practice, the delay is usually smaller by several orders of magnitude than the period T of the sawtooth profile. Similarly, the beat frequency df is normally much smaller than the frequency span $f_{\max}-f_{\min}$.

In the FMCW radar, a wave whose frequency is swept linearly in time, as shown in Figure 1, is transmitted toward the target and then the wave returning from the target is mixed with a copy of the transmitted wave. Since propagation introduces a delay, the two waves will have different frequencies, which will result in a beat signal when mixed. The linearity of the frequency sweep makes sure that the beat frequency is constant during the overlapping part of the sweep. The beat frequency depends on the delay, which in turn depends on the distance to the target; thus, the distance can be determined by measuring the beat frequency.

In our proposed technique, the FMCW radar principle is used as described, except that the frequency modulation is not applied to the carrier, as is usual with FMCW radar systems, but to a subcarrier obtained by amplitude modulation.

**Figure 2:**
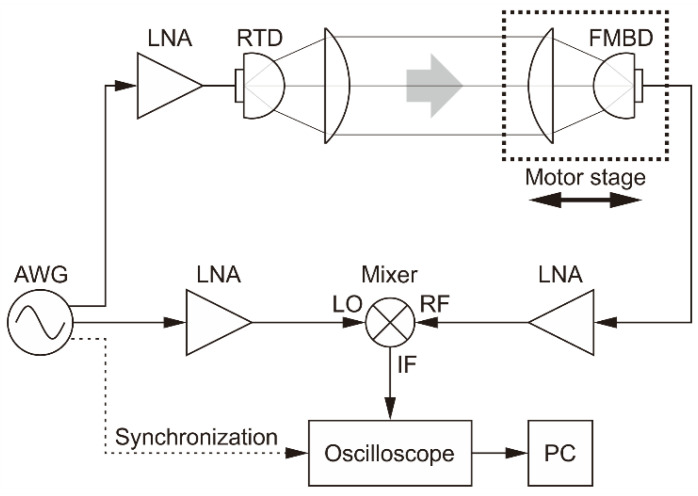
Schematic of the electrical and in-line optical setup. AWG: arbitrary waveform generator, LNA: low-noise amplifiers for power level adjustment and detected signal enhancement. In this particular optical configuration, the radar only measures the propagation distance between the terahertz source and detector. To change the propagation distance, the detector and its lens (marked by a dotted rectangle) are moved together as a unit. Auxiliary circuit elements such as power supplies, a bias tee, etc. are omitted for simplicity.

To illustrate the measurement principle, we show in Figure 2 a schematic of the basic setup that we used to experimentally verify the theory. In this most elementary in-line optical configuration, there is no target; the radar simply measures the propagation time, which we can convert into propagation distance. The propagation occurs on two separate paths, both starting at the arbitrary waveform generator (AWG) that produces the sweep signal and ending at the frequency mixer that outputs the beat signal: the reference path goes directly from the generator to the mixer’s LO (local oscillator) input through cables and an amplifier, whereas the measurement path includes the distance in air between the terahertz-wave source and the detector, in addition to propagating through cables and being amplified several times before reaching the RF (radio frequency) input of the mixer. The time measured by the radar by determining the frequency of the beat signal obtained at the IF (intermediate frequency) port of the mixer is the difference between the delays caused by propagation on these two paths.

Mathematically, a linear frequency sweep is a signal whose instantaneous frequency increases linearly from $f_{\min}$ to $f_{\max}$ in the period T:

(1) $$f\left(t\right)=f_{\min}+\left(f_{\max}-f_{\min}\right)\frac{t}{T}$$

where time t starts from 0 and ends at T. To generate the waveform that has this frequency profile, we can define the instantaneous frequency as being proportional to the time derivative of the phase:

(2) $$f\left(t\right)=\frac{1}{2\pi }\;\frac{d\varphi }{dt}$$

By plugging Equation (1) into (2) and integrating, the phase of the waveform that needs to be calculated is obtained:

(3) $$\varphi \left(t\right)=\varphi_{0}+2\pi f_{\min}t+2\pi \frac{f_{\max}-f_{\min}}{T}\cdot \frac{t^{2}}{2}$$

where $\varphi_{0}$ is an initial phase that can be given any value; we set it to zero. We choose a sinusoidal function of an arbitrary amplitude $A_{\mathrm{AWG}}$ and arrive at the following formula for the AWG output voltage, $V_{\mathrm{AWG}}$, which we used to generate the waveforms for our experiments:

(4) $$V_{\mathrm{AWG}}\left(t\right)=A_{\mathrm{AWG}}\;\cos\left(2\pi f_{\min}t+2\pi \frac{f_{\max}-f_{\min}}{T}\cdot \frac{t^{2}}{2}\right)$$

From the AWG, the signals propagate on the reference path and the measurement path, each of which introduces a delay, $t_{\mathrm{ref}}$ and $t_{\mathrm{meas}}$, respectively, until the signals meet again in the frequency mixer. At the mixer LO and RF inputs, the signals can be written as follows:

(5) $$V_{\mathrm{LO}}\left(t\right)=A_{\mathrm{LO}}\;\cos\left(2\pi f_{\min}\left(t-t_{\mathrm{ref}}\right)+2\pi \frac{f_{\max}-f_{\min}}{T}\cdot \frac{{\left(t-t_{\mathrm{ref}}\right)}^{2}}{2}\right)$$

(6) $$V_{\mathrm{RF}}\left(t\right)=A_{\mathrm{RF}}\;\cos\left(2\pi f_{\min}\left(t-t_{\mathrm{meas}}\right)+2\pi \frac{f_{\max}-f_{\min}}{T}\cdot \frac{{\left(t-t_{\mathrm{meas}}\right)}^{2}}{2}\right)$$

where the LO and RF subscripts relate to the respective mixer input, and the signal amplitudes $A_{\mathrm{LO}}$ and $A_{\mathrm{RF}}$ depend on the various gains and losses occurring on each signal path.

The mixer multiplies $V_{\mathrm{LO}}$ and $V_{\mathrm{RF}}$, and the product becomes accessible at its IF output as the following $V_{\mathrm{IF}}$ voltage:

(7) $$V_{\mathrm{IF}}\left(t\right)=k\;V_{\mathrm{LO}}\left(t\right)\;V_{\mathrm{RF}}\left(t\right)$$

where k is a coefficient related to the conversion loss of the mixer. The IF signal then becomes

(8) $$V_{\mathrm{IF}}\left(t\right)=A_{\mathrm{IF}}\;\cos\left(2\pi \left(f_{\max}-f_{\min}\right)\frac{t_{\mathrm{meas}}-t_{\mathrm{ref}}}{T}t-\Phi \right)$$

in which all the time-independent terms obtained by multiplying the two signals were grouped in the constant phase Φ; the high-frequency term corresponding to the sum of the two frequencies was removed and only the low-frequency beat signal was retained. The IF signal amplitude $A_{\mathrm{IF}}$ is proportional to the product of $A_{\mathrm{LO}}$ and $A_{\mathrm{RF}}$ and is generally a slowly varying function of the instantaneous modulation frequency, particularly because the RTD output is more efficiently modulated at lower frequencies.

The result of this calculation is that the frequency of the IF signal is

(9) $$f_{\mathrm{IF}}=\left(f_{\max}-f_{\min}\right)\frac{t_{\mathrm{meas}}-t_{\mathrm{ref}}}{T}$$

which gives the propagation time difference as

(10) $$t_{\mathrm{meas}}-t_{\mathrm{ref}}=\frac{T}{f_{\max}-f_{\min}}f_{\mathrm{IF}}$$

From here, the distance to the target can be calculated, taking into account the optical configuration. In the usual situation, where the terahertz wave makes a round trip to and from the target and the propagation takes place in air, the distance to the target becomes

(11) $$d_{\mathrm{target}}=\frac{T}{f_{\max}-f_{\min}}f_{\mathrm{IF}}\frac{c}{2\;n_{\mathrm{air}}}$$

where c is the speed of light in vacuum, and $n_{\mathrm{air}}$ is the refractive index of air (about 1.0003 at terahertz frequencies). The multiplier 2 at the denominator accounts for the round trip; it must be replaced with 1 for the in-line optical setup in Figure 2.

To measure the beat frequency $f_{\mathrm{IF}}$, the IF signal is recorded and processed by calculating its Fourier transform. When the target consists of parts placed at various distances from the radar, each of them will show up as a separate peak in the Fourier transform. The FMCW radar can measure all those distances simultaneously. However, since the IF signal is necessarily limited in time—only one period T can be used, as the phase is reset at the beginning of each period—the resolution of measuring the beat frequencies, that is, the ability of resolving the various peaks, is limited to approximately the inverse of T:

(12) $$\delta f_{\mathrm{IF}}≅\frac{1}{T}$$

Consequently, the distances can also be measured only with a limited resolution, given by the following equation:

(13) $$\delta d_{\mathrm{target}}≅\frac{1}{f_{\max}-f_{\min}}\;\frac{c}{2\;n_{\mathrm{air}}}$$

If there is only one distance to be measured, such as in the case of a small flat target reflecting the beam back to the radar, this resolution is almost meaningless, since the error of measuring the position of the single peak, which depends on the signal-to-noise ratio and the processing method, can be several orders of magnitude smaller than the resolution.

## 3. Experimental Verification

To verify experimentally the principle of our radar, we used two optical configurations. First, the in-line setup already shown schematically in Figure 2 was used, with which we confirmed the ability of the radar to measure propagation distances. Second, a reflection setup was used, in which the terahertz beam travels to a target and returns.

The emission of the RTD oscillator is at 511 GHz and has a peak power of around 10 µW. The output amplitude is modulated by first lowering the bias voltage slightly from the value that produces the peak power, so as to make the output power sensitive to variations of the bias voltage, and then adding the modulation signal to the bias voltage by using a bias tee. In the particular case of the subcarrier FMCW radar, the modulation signal is a chirp, for which we used an AWG whose playback memory was filled with a linearly sweeping sine from 3.4 to 6.4 GHz; these frequencies can be changed, but are ultimately limited by other components in the circuit. The period of the sweep signal is around 2 µs. On a second channel of the AWG, we set up a synchronization pulse to be emitted at the beginning of each frequency sweep; this pulse is needed for triggering the oscilloscope and thus allowing it to average a series of waveforms so as to improve the signal-to-noise ratio; in most of our measurements, we averaged 1024 waveforms and obtained a signal-to-noise ratio around 20 dB. The total time for recording and processing the IF signal is around 80 s; most of this time is taken by the oscilloscope for performing the digital calculations involved in averaging the signal.

The RTD oscillator emits a terahertz beam that is first narrowed by a silicon lens attached to the RTD chip and then collimated by a plastic lens. After propagating through air, the beam is focused by a second plastic lens onto a detector. We used a Fermi-level-managed-barrier-diode (FMBD) detector [17], which in our particular setup turned out to have a signal-to-noise ratio about 10 dB better than another high-speed terahertz-wave detector that we tried, namely a Schottky-barrier-diode (SBD) detector.

## 4. In-Line Setup

To assess the radar’s ability to measure the propagation distance precisely, for the in-line configuration shown in Figure 2 we placed the detector and its focusing lens on a computer-controlled motor stage with a movement range of 200 mm.

**Figure 3:**
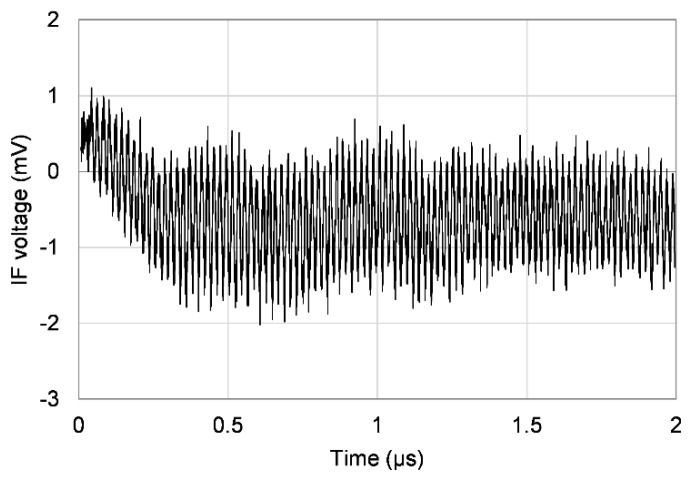

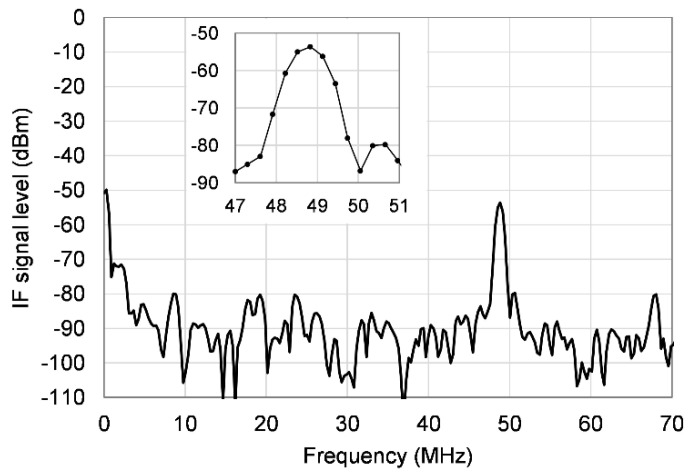
An example of the IF beat signal. Top: the IF signal recorded by the oscilloscope. The period is 2 µs and the modulation frequency is swept from 3.4 to 6.4 GHz. Bottom: the Fourier transform of the IF signal, showing a peak near 49 MHz; the inset expands the region around the peak. This figure is for illustration only and does not correspond to any of the other results in the paper; the signal shown here was recorded after a series of improvements increased the signal-to-noise ratio.

The signal from the FMBD detector is amplified first by an internal trans-impedance amplifier and then by an external low-noise amplifier. This signal is connected to the RF input of the mixer. The LO input is a copy of the frequency sweep that is sent to the RTD. The mixer’s IF output provides the beat signal, which is sent to the oscilloscope. The signal is Fourier transformed in the oscilloscope and the result is transferred to a computer for subsequent peak detection and distance calculation; a LabVIEW program communicates with the oscilloscope, performs all the necessary calculations, and controls the motor stage. Figure 3 shows an example of the IF signal and the corresponding Fourier transform.

The details of the data processing play a crucial role in the final precision of the radar. Before the Fourier transform, the oscilloscope multiplies the averaged IF signal with a Hann (raised cosine) window function and zero-pads it for the fast Fourier transform (FFT) calculation. The LabVIEW program searches the FFT result for a peak in the range of interest and then fits a Gaussian function to the strongest three points of the detected peak, so as to determine the IF frequency with a resolution much finer than the bin spacing of the Fourier transform.

The selection of the windowing function and of the fitting function determines the overall ranging precision. We chose the Gaussian function for peak fitting primarily due to its simplicity—with the signal recorded in dBm units, the Gaussian becomes a parabola—and then chose the windowing function from those available in the oscilloscope (Hann, flat top, Blackman-Harris, Hamming); in our particular conditions, the pairing of the Hann window with the Gaussian fitting turned out to achieve the smallest ranging error. Mathematically, this method of finding the peak position is still not ideal, as even in noiseless simulations we found that it produces residual errors; we are currently looking into ways to reduce those errors even further. In a separate research subject on a terahertz-wave radar based on the optical coherence tomography (OCT) principle, which also relies on finding peaks in Fourier transform data (not published yet), we found that using a Gaussian windowing in conjunction with the Gaussian peak fitting can lead to a reduction in the ranging errors if properly optimized.

**Figure 4:**
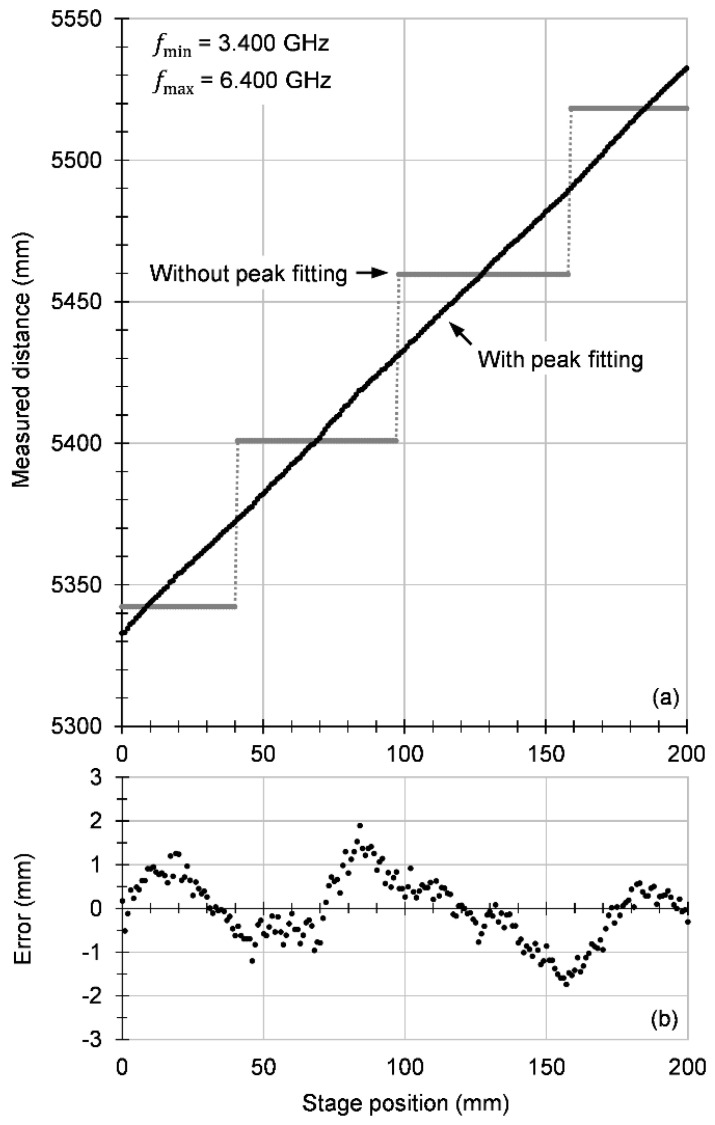
(**a**) Propagation distance measured by the radar in the in-line optical configuration as a function of the motor stage position. Distances calculated without Gaussian peak fitting are shown for reference. (**b**) Error of the distance measurement, defined as the differences between the measured distances and a linear fit of the data with the slope fixed to 1. The standard deviation of the errors is 0.73 mm.

Figure 4 shows the result of a measurement confirming the measurement principle. When the motor stage takes the detector away from the source, the distance measured by the radar increases as expected, with a slope approximately equal to 1. The radar’s ability to measure absolute distances is illustrated by the ordinate values: when the stage is at 0, the propagation time in the measurement arm, including propagation in cables and air, minus the propagation time in the reference arm (cables only), converted into propagation distance in air, is about 5332 mm. This can be checked separately by measuring the cable lengths, the propagation speed in cables, and the distances between the optical components, but the overall error of estimating the total distance is in the order of at least a few centimeters, which is much worse than the precision provided by the radar, such that an accurate confirmation cannot be obtained independently.

The slope of the data in Figure 4a was determined by linear fitting and turned out to be slightly different from 1, namely 0.996, which for our 200 mm movement represents a drift of 0.8 mm. We believe this was caused by a combination of factors, of which one is the fact that when the stage moves, the cable carrying the signal away from the FMBD changes its curvature, such that the length seen by the signal changes slightly with the stage position; another factor is the waviness of the measured data, which is discussed below. The motor stage is specified to have a positioning error below 15 µm, such that we do not expect it to contribute significantly to the measurement error.

Figure 4b shows the measurement errors. Since the absolute distance, including inside cables, cannot be measured by other means, we defined the measurement error as the difference between the distance measured by the radar and a linear fit of the whole measurement set, while making sure the slope of the linear fit is fixed to exactly 1 and only the intercept is allowed to vary. With this definition, the errors turn out to have a standard deviation of about 0.73 mm.

There are two main components visible in the error plot. One consists of random fluctuations and is caused by the noise in the system; the difference between the noise floor in the Fourier transform and the peak is only around 20 dB. Another component is a wavy, somewhat periodic, pattern. This could have several causes: the periodic character might arise from some interference effect in the optical setup and from the residual error of our fitting routine. However, we attribute the main part of the error to a pattern of small but systematic peaks in the Fourier transform, which we call ‘ghosts’. They are likely caused by small reflections at the many connection points in the electrical circuit as well as at the several lens surfaces in the optical path. The chirp signal reflects between pairs of such points and produces weak copies of the original chirp with a wide variety of time delays. Through mixing, these weak chirps generate a correspondingly wide variety of beat signals, each causing a small peak to appear at various points in the Fourier transform. When the main peak comes near such a weak peak, the position of the main peak calculated by fitting is slightly shifted toward the weak peak, producing an error in the measured propagation time.

## 5. Reflection Setup

**Figure 5:**
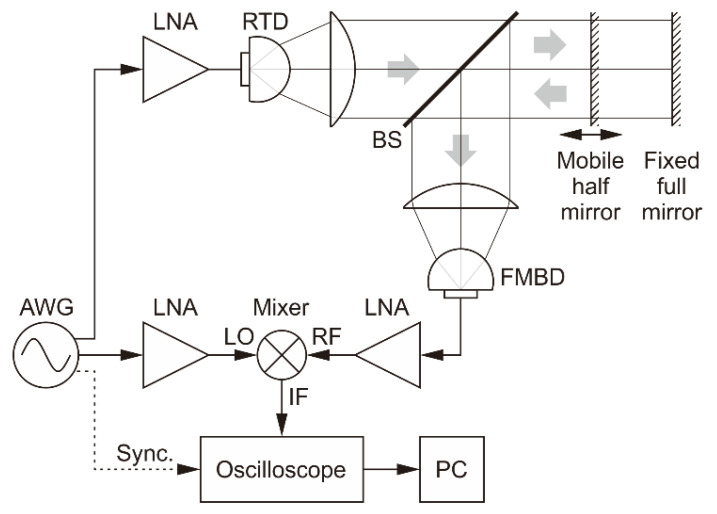
Schematic of the experimental setup configured in reflection. The beam splitter (BS) allows a normal incidence on the target, which in this case consists of a half mirror and a full mirror.

While the experiments with the in-line setup can be considered sufficient to confirm the measurement principle, we wanted to go one step further and demonstrate the method in a typical radar configuration, wherein the beam is reflected by a target. This also allowed us to test whether the radar can measure more than one distance simultaneously. For that purpose, we used one half mirror (a thin high-resistivity silicon plate with a transmission coefficient of roughly 50%) and one full mirror (a gold-coated glass plate with a reflecting coefficient close to 100%) as targets. The beam arriving at the half mirror is partially reflected and partially transmitted toward the full mirror; with this optical arrangement, the radar sees two targets at different distances. The half mirror was mounted on the same motor stage used for the in-line measurements; moving the half mirror in a precise manner allows the evaluation of the ranging error. The modified experimental setup is shown in Figure 5.

To achieve the normal incidence of the beam on the mirrors used as targets, we used a beam splitter made of a thin plate of high-resistivity silicon, whose thickness was chosen such that it has a 50% transmission and 50% reflection (the absorption is negligible) at the 45° incidence and at the particular orientation of the polarization plane in our setup. This precaution optimizes the total optical efficiency, as the beam is once transmitted and once reflected by the beam splitter. Nevertheless, the use of this beam splitter reduces the available power of the terahertz wave to 25%; the remaining 75% is lost in beams that are sent outside the optical setup or toward the RTD source. Using waveplates can recover much of that loss, but complicates the setup, and we chose not to try it at this time.

In addition to changing the optical configuration, we also tested a number of signal-to-noise improvement techniques:

1. Bandwidth maximization. We increased $f_{\max}$ from 6.4 to 10.5 GHz, which extends the frequency span from 3 to 7 GHz and has a significant beneficial effect on the distance measurement precision; just by extending the sweep span, the error reduced from the 0.73 mm reported above to 0.36 mm in the in-line setup. In addition, the theoretical resolution improves by a factor larger than 2. The maximum modulation frequency is currently limited by the internal amplifier of the FMBD detector; as a new FMBD with a bandwidth of 34 GHz has recently become available, we intend to use it in our future investigations.
2. Frequency-dependent amplitude adjustment. Several components in the electrical circuit have frequency-dependent gain characteristics. The RTD oscillator, in particular, includes an intrinsic RLC low-pass filter on the biasing connection, which means that the modulation amplitude of its output depends rather strongly on the modulation frequency, such that higher frequencies produce weaker signals. To some extent, this can be compensated by adjusting the amplitude of the AWG signal as a function of the frequency. In our experiments, however, the benefit of making this adjustment was minimal.
3. Ghost avoidance. The systematic peaks in the Fourier transform were found to have higher intensities and densities in some distance ranges than in others. To decrease their influence on the measurement, we extended some of the cables such that the peak corresponding to our target falls in a quieter range of distances, with fewer and weaker ghost peaks.
4. Background subtraction. Since the ghost peaks cannot be completely avoided, we also applied a background subtraction method, which consists in making one measurement without any target and recording the corresponding Fourier transform as background, which was then subtracted from subsequent data. However, the random noise level is just a few dB below the ghost level, and as such, subtracting the background has only a limited beneficial effect.

**Figure 6:**
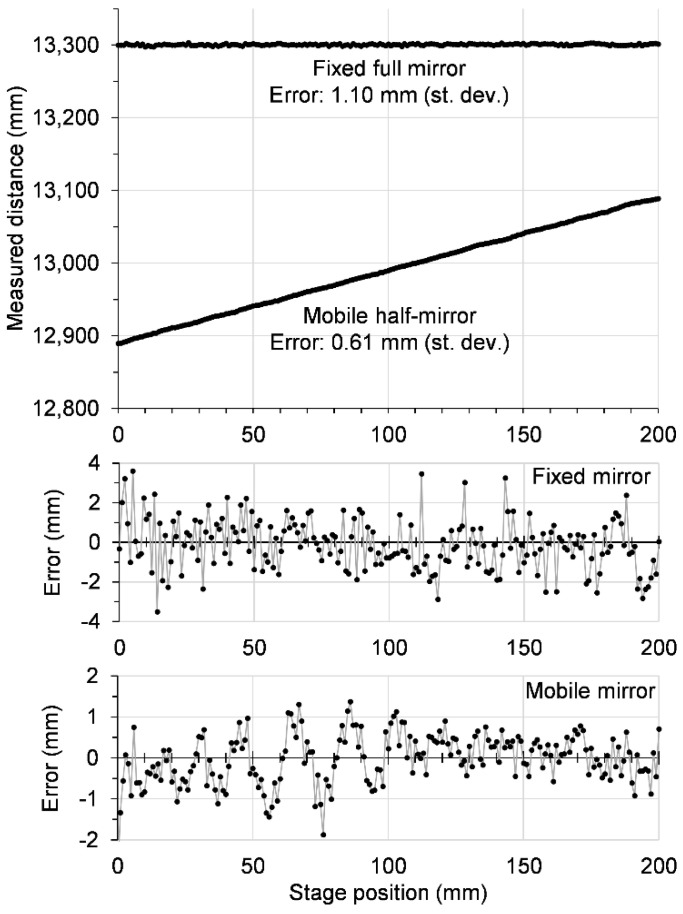
Measurement using the setup shown in Figure 5, with the mobile full mirror moved in 1-mm steps over a distance of 200 mm. The top graph shows the measured distance as a function of the motor stage position. The measurement error for each target is shown in the two bottom plots, where the error is defined as the difference between the measured data and a linear fit with slopes 0 and 1 for the fixed and mobile mirrors, respectively.

Figure 6 shows a typical result obtained using the reflection optical setup and two targets. The improvements detailed above proved to have a more positive effect than the loss of signal caused by the beam splitter, such that the ranging error for the mobile half mirror turned out to be 0.61 mm (better than the 0.73 mm obtained with the full mirror in the in-line setup), and there was still enough signal-to-noise room to also detect the weaker signal of the fixed full mirror, albeit with a larger error, of 1.10 mm.

## 6. Conclusions

We have proposed a new radar technique, based on the classical FMCW method, but where the frequency modulation is applied to an amplitude-modulation subcarrier. We demonstrated this technique with a terahertz-range RTD oscillator, for which using a subcarrier is at present more practical than precisely modulating the carrier frequency. The experiments with our subcarrier FMCW radar system show that it can measure absolute distances with precisions below 1 mm; this level of precision was obtained solely by fitting the peaks with Gaussian functions, without processing the phase available in the Fourier transform.

As expected from the measurement principle, the simultaneous ranging of two targets was shown to be possible, and nothing suggests that measuring more distances could not be achieved, except that more targets would mean weaker, and thus harder to detect, peaks in the Fourier transform. The theoretical resolution, as calculated from Equation (13), should be about 21 mm for a bandwidth of 7 GHz; we have not yet attempted to verify this experimentally.

RTD oscillators are expected to be developed in the near future with output powers in the milliwatt order, that is, about 100 times more powerful than the device we used. Also, RTD oscillators with particular structures have been shown to allow modulation up to as much as 30 GHz [18]. A device having both high output power and wide modulation band would improve greatly both the signal-to-noise ratio and the ranging precision of the subcarrier FMCW radar, as well as bring the measurement closer to real time. We are currently attempting to achieve real-time ranging by optimizing the measurement parameters (biasing conditions, frequency bandwidth, sweep duration, etc.) and signal processing while still using the same 10 µW RTD.

Although research on terahertz-wave radar techniques is still in its infancy and much work needs to be done to bring these ideas into practical applications, we believe there is great potential in using terahertz waves for ranging and ultimately 3-dimensional imaging in environments where other types of electromagnetic waves would be unusable or less effective.
